# The EAT-Lancet diet, genetic susceptibility and risk of atrial fibrillation in a population-based cohort

**DOI:** 10.1186/s12916-023-02985-6

**Published:** 2023-07-28

**Authors:** Shunming Zhang, Anna Stubbendorff, Ulrika Ericson, Per Wändell, Kaijun Niu, Lu Qi, Yan Borné, Emily Sonestedt

**Affiliations:** 1 School of Public Health, Xi’an Jiaotong University Health Science Center, Xi’an, Shaanxi China; 2 Nutritional Epidemiology, Department of Clinical Sciences Malmö, Lund University, Jan Waldenströms Gata 35, 21428 Malmö, Sweden; 3 Diabetes and Cardiovascular Disease-Genetic Epidemiology, Department of Clinical Sciences Malmö, Lund University, Malmö, Sweden; 4 Department of Neurobiology Care Sciences and Society, Division of Family Medicine and Primary Care, Karolinska Institutet, Huddinge, Sweden; 5 Nutritional Epidemiology Institute and School of Public Health, Tianjin Medical University, Tianjin, China; 6 Department of Epidemiology, School of Public Health and Tropical Medicine, Tulane University, New Orleans, LA USA; 7 Department of Nutrition, Harvard T.H. Chan School of Public Health, Boston, MA USA

**Keywords:** Atrial fibrillation, EAT-Lancet diet, Sustainable diet, Genetic risk score

## Abstract

**Background:**

The EAT-Lancet Commission proposed a global reference diet with both human health benefits and environmental sustainability in 2019. However, evidence regarding the association of such a diet with the risk of atrial fibrillation (AF) is lacking. In addition, whether the genetic risk of AF can modify the effect of diet on AF remains unclear. This study aimed to assess the association of the EAT-Lancet diet with the risk of incident AF and examine the interaction between the EAT-Lancet diet and genetic susceptibility of AF.

**Methods:**

This prospective study included 24,713 Swedish adults who were free of AF, coronary events, and stroke at baseline. Dietary habits were estimated with a modified diet history method, and an EAT-Lancet diet index was constructed to measure the EAT-Lancet reference diet. A weighted genetic risk score was constructed using 134 variants associated with AF. Cox proportional hazards regression models were applied to estimate the hazard ratio (HR) and 95% confidence interval (CI).

**Results:**

During a median follow-up of 22.9 years, 4617 (18.7%) participants were diagnosed with AF. The multivariable HR (95% CI) of AF for the highest versus the lowest group for the EAT-Lancet diet index was 0.84 (0.73, 0.98) (*P* for trend < 0.01). The HR (95% CI) of AF per one SD increment of the EAT-Lancet diet index for high genetic risk was 0.92 (0.87, 0.98) (*P* for interaction = 0.15).

**Conclusions:**

Greater adherence to the EAT-Lancet diet index was significantly associated with a lower risk of incident AF. Such association tended to be stronger in participants with higher genetic risk, though gene-diet interaction was not significant.

**Supplementary Information:**

The online version contains supplementary material available at 10.1186/s12916-023-02985-6.

## Background

Atrial fibrillation (AF) is the most common cardiac arrhythmia in clinical practice and is affected by genetic and environmental factors [1]. AF confers increased risks of stroke, heart failure, and approximate doubling of all-cause mortality [2, 3]. According to the European Society of Cardiology AF guidelines, 43.6 million individuals had prevalent AF globally in 2016 [4]. The incidence of AF in Sweden was 4.0 per 1000 person-years in 2011–2012 and increased with age [5]. Therefore, identifying modifiable risk factors (e.g., diet) for AF may play an important role in reducing the global public health burden associated with AF.

While associations between various dietary patterns and AF have been examined [6–12], evidence regarding the EAT-Lancet diet with AF is still lacking. The EAT-Lancet diet was proposed in 2019 by the EAT-Lancet Commission, considering both globally environmentally sustainable and human health [13]. Compared with healthful plant-based diets that completely exclude beneficial animal foods such as dairy products and fish [14], the EAT-Lancet diet emphasizes a high intake of healthy plant foods (whole grains, vegetables, fruits, legumes, and nuts), moderate intake of fish, and a low intake of meat, dairy products, tubers and starchy vegetables, added fats, and sugars [13]. Thus, this diet may be easier to follow for most meat-preferring populations. However, knowledge about the health effects in different populations following the EAT-Lancet diet is sparse because individual dietary variables in relation to risks of chronic diseases do not well reflect the overall food combination effect. In addition, different populations may have different eating habits and lifestyles, which affects the health benefits of the EAT-Lancet diet. In this regard, an EAT-Lancet diet index has been developed and validated in our recent work and showed an inverse association with mortality [15]. However, no study has investigated the association between the EAT-Lancet diet and the risk of AF. Furthermore, genetic predisposition plays an important role in the development of AF [16]. Studies have suggested that genetic predisposition to AF modifies the association of environmental factors (e.g., sleep) with the risk of AF [17]. However, whether the genetic predisposition to AF can be offset by adopting the EAT-Lancet diet is not known.

In the current study with nearly 30 years of follow-up, we examined the associations of the EAT-Lancet diet index with the risk of AF. We hypothesized that the EAT-Lancet diet index would be associated with a lower risk of AF. In addition, we evaluated the interaction between the EAT-Lancet diet index and genetic susceptibility on the risk of AF and hypothesized that the importance of the EAT-Lancet diet index would differ between those with low, medium, and high genetic susceptibility to AF.

## Methods

### Study population

This prospective cohort study utilized data from the Malmo Diet and Cancer Study (MDCS) [18]. The MDCS was established in 1991–1996. All men born between 1923 and 1945 and all women born between 1923 and 1950 living in Malmö were invited to participate in the study. All participants completed a series of lifestyle assessments and underwent physical examinations at baseline (1991–1996). When recruitment closed, a total of 30,446 completed at least one part of the baseline examination, and 28,098 participants completed dietary assessment, lifestyle questionnaire, and anthropometric measurements. The study protocol was approved by the Ethical Committee at the Medical Faculty at Lund University (approval number: LU 51–90). Written informed consent was obtained from all participants.

**Fig. 1:**
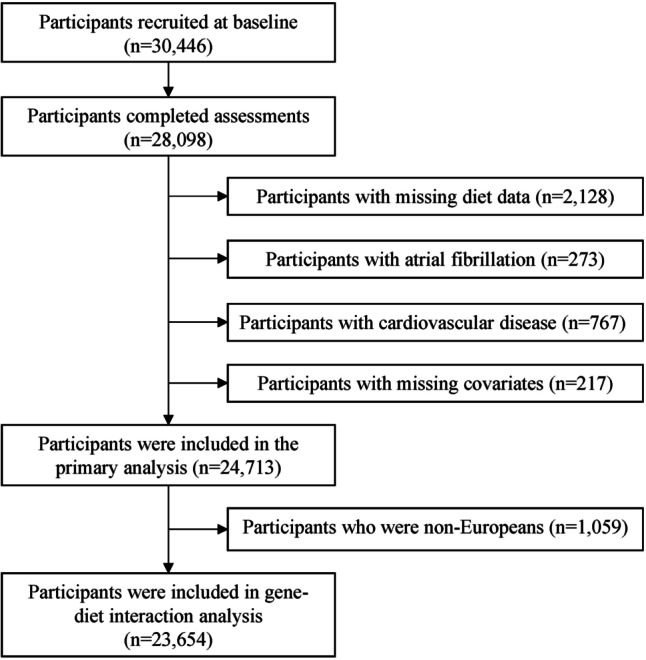
The flowchart of study participant selection

In the current study, we excluded participants with baseline examination during 1991 because of the lack of data on legumes separately (*n* = 2128). In addition, we further excluded those who had prevalent AF and/or atrial flutter (AFL) (*n* = 273) or cardiovascular diseases (including coronary events or stroke, *n* = 767) at baseline since these diseases might cause significant dietary changes. Moreover, we excluded those who had missing data on covariables (*n* = 217), including missing leisure time physical activity (*n* = 135), body mass index (BMI) (*n* = 30), education (*n* = 46), and smoking (*n* = 6). After these exclusions, the final analytic sample included 24,713 participants. Non-European individuals (*n* = 1059) based on genetic data were also excluded from the gene-diet interaction analysis. Figure 1 shows the flowchart of the sample selection. The reporting of the study was conducted following the Strengthening the Reporting of Observational Studies in Epidemiology (STROBE) guidelines [19] and the STROBE-nut guidelines [20].

### Assessment of dietary intake

At baseline, dietary intake was assessed by combining a 7-day food diary, a 168-item food frequency questionnaire (FFQ), and an in-person interview [21]. The 7-day food diary recorded cooked/main meals, cold beverages, and dietary supplements. The FFQ collected the frequency and portion size information of foods habitually consumed over the past year (not overlapping with the food diary). The interview was conducted to attain information on cooking habits and portion sizes in the food diary. Each food intake amount (g/day) was calculated by summing the information from the food diary/interview and the FFQ. Total energy and nutrient intake data were generated from the intake amounts, using the Swedish Food Database PC KOST2-93 of the Swedish National Food Administration. The reproducibility and validity of the dietary assessment method have been documented in detail elsewhere [22, 23]. The method performed reasonably well when ranking individuals according to their usual dietary intake.

### Construction of the EAT-Lancet diet index

Based on the EAT-Lancet Commission recommendations [13], we recently developed an EAT-Lancet diet index [15, 24, 25]. The criterion for the creation of the EAT-Lancet index has been described in detail previously [15]. In brief, the index consisted of 14 food groups, including 7 emphasized foods (vegetables, fruits, unsaturated oils, legumes, nuts, whole grains, and fish) and 7 limited foods (beef and lamb, pork, poultry, eggs, dairy, potatoes, and added sugar). Scores of each food component ranged from 0 (lowest adherence) to 3 (highest adherence). The EAT-Lancet diet index score ranged from 0 to 42 with higher scores indicating more adherence to the EAT-Lancet reference diet. Adherence was categorized into five groups: ≤ 13 points (the reference), 14–16 points, 17–19 points, 20–22 points, and ≥ 23 points to make each group as similar as possible in size to ensure the same score interval between the groups and to have adequate numbers of AF cases in each group [15].

### Ascertainment of AF

As in previous studies [17, 26], the outcome of AF included AF and AFL events, given the close interrelationship of these diseases [27]. Prevalent and incident AF cases were retrieved from the Swedish National Inpatient Register and the Swedish Causes of Death Register [26]. The National Inpatient Register has been operating in Malmö since 1969 and has been compulsory nationwide since 1987. The Causes of Death Register includes diagnoses from death certificates since 1952, regardless if the death occurred outside of Sweden. The AF cases were ascertained using the International Classification of Diseases (ICD) codes 427.92 (ICD-8, used up to 1986), 427D (ICD-9, used between 1987 and 1996), and I48 (ICD-10, used from 1997) [26]. The validation study of AF diagnoses in registers showed that the validity of AF cases in MDCS is high (> 97%), and case misclassification of AF is small, indicating the feasibility of use in epidemiological research [26]. All participants were followed up from the completion date of the baseline survey until the date of the first diagnosis of AF, death, migration from Sweden, or 31 December 2018, whichever came first. The rate of loss to follow-up was less than 1% (*n* = 198).

### Genetic risk score (GRS)

The Illumina GSA v1 genotyping array was used for genotyping by Regeneron Genetics Center (Tarrytown, NY). Genotype imputation was performed using the Michigan Imputation Server with a reference panel of the Haplotype Reference Consortium (HRC r1.1) [28]. Details of quality control of genetic data have been published elsewhere [29]. A weighted GRS was calculated based on the 134 single nucleotide polymorphisms (SNPs) that were identified from the most recent meta-analysis of genome-wide association study of the risk of AF [16], which is consistent with a recent study [17] (Additional file 1: Table S1). Each SNP was weighted by its effect size (*β* coefficient), and the *β* coefficients were derived from the genome-wide meta-analysis [16]. The weighted genetic risk score was constructed using the following equation: GRS = (*β*_1_ × SNP_1_ + … + *β*_134_ × SNP_134_) × (134/sum of the *β* coefficients), where SNP indicates the risk allele number of each SNP (0, 1, or 2). The calculated GRS of AF ranged from 76.6 to 128.7, and Additional file 1: Fig. S1 shows the distribution of AF risk in the population (*n* = 23,654). A higher score represents a higher genetic predisposition to AF. The GRS was divided into quintiles to stratify participants into low (quintile 1), intermediate (quintiles 2–4), and high (quintile 5) genetic risk.

### Assessment of covariates

Age and sex of the study participants were collected via their civic registration numbers. Information on smoking status (current smoker, ex-smoker, and non-smoker) and educational level was collected through a self-administrated questionnaire. Leisure time physical activity was estimated based on 17 common activities (e.g., jogging, cycling, and swimming) and was expressed as metabolic equivalent task (MET) hours/week. Then, leisure time physical activity was divided into five groups: 0–7.5, 7.5–15, 15–25, 25–50, and > 50 MET-hour/week. Alcohol consumption was divided into sex-specific quintiles based on the current consumption of the participants, as reported in the 7-day food diary. Individuals reporting no alcohol intake the previous year in the lifestyle questionnaire and no alcohol in the 7-day food diary were categorized as zero-consumers of alcohol. A change in coding routine in 1994 [21], where the dietary interview was shortened from 60 to 45 min, resulted in the variable “method (dietary assessment version).” The variable “season” refers to the time of year the baseline diet data collection took place (summer, autumn, winter, and spring).

Anthropometric measurements (height and weight) were conducted by trained nurses following a standardized procedure. BMI (kg/m^2^) was calculated using height and weight. Baseline diabetes was ascertained by a self-reported diabetes diagnosis, self-reported diabetes medication, or information from the national and local registries. Blood pressure was measured using a mercury column sphygmomanometer after 10 min of rest in a supine position. Hypertension was defined as systolic blood pressure ≥ 140 mmHg and/or diastolic blood pressure ≥ 90 mmHg or taking antihypertensive drugs. The use of lipid-lowering medication was obtained through the questionnaire.

### Statistical analysis

Baseline characteristics of the study participants were expressed as means ± standard deviation (SD) for continuous variables and as *n* (%) for categorical variables. Chi-squared was used for categorical variables and one-way analysis of variance for continuous variables. Cox proportional hazards regression models were used to assess the associations of the EAT-Lancet diet index and its components with AF risk, with time-on-study on the time scale. The proportional hazards assumption was checked by including an interaction term between the EAT-Lancet diet index and log survival time, and no violation was found (*P* = 0.45). The results were presented as hazard ratios (HRs) and 95% confidence intervals (CIs). Model 1 was adjusted for age, sex, dietary assessment version (method), season, and total energy intake. Model 2 was adjusted for variables in model 1 plus leisure time physical activity, alcohol consumption, smoking status, and educational level. Model 3 was adjusted for variables in model 2 plus BMI. Model 4 was additionally adjusted for diabetes, hypertension, and the use of lipid-lowering medication. The *P* for trend was calculated by assigning the categories of the EAT-Lancet diet index as an ordinal variable (≤ 13: 1, 14–16: 2, 17–19: 3, 20–22: 4, and ≥ 23: 5).

In order to examine the robustness of the results, we conducted a series of sensitivity analyses. First, considering that diabetes might cause important diet changes, we excluded those with prevalent diabetes (*n* = 1044). Second, to better reflect usual dietary intakes, we excluded potential energy misreporters and individuals that had reported a substantial eating habit change in the past (*n* = 8862). Third, to minimize the effect of potential reverse causation, we examined such associations by excluding AF cases ascertained within the first 2 (*n* = 89) or 5 years (*n* = 293) of follow-up. Furthermore, we performed subgroup analyses to assess the potential effect modifications by sex, age, BMI, leisure time physical activity, alcohol habits, education level, and smoking status. Potential interactions between these stratification variables and the EAT-Lancet diet index were assessed using the likelihood ratio test by comparing models with and without the interaction terms.

We further performed a stratified analysis by AF-GRS (low, medium, and high) to assess the association of the EAT-Lancet diet index with the risk of AF among participants with different genetic risks. In this analysis, the EAT-Lancet diet index was entered into the model as a continuous variable (one SD increase for the EAT-Lancet diet index). To evaluate the interaction between AF-GRS and the EAT-Lancet diet index on AF risk, the multiplicative interaction term of the EAT-Lancet diet index (continuous) and AF-GRS (low, medium, and high) was added to the Cox proportional hazards model.

All statistical analyses were performed in the SAS software version 9.4 (SAS Institute Inc., Cary, NC, USA). Two-sided *P* values < 0.05 were deemed as statistically significant.

## Results

Table 1 shows the baseline characteristics of the study participants. The mean ± SD age of participants was 58.0 (7.7) years. Of the 24,713 participants, 9348 (37.8%) were men. Overall, participants with higher EAT-Lancet diet scores were less likely to be men, had high education degree, were more physically active, were less likely to be current smokers, were more likely to have diabetes, tended to use lipid-lowering and antihypertensive drugs, and consumed less total energy.

**Table 1:** Baseline characteristics of the study participants, overall and by categories of the EAT-Lancet diet index (*n* = 24,713)^1^

Characteristics	Total	Categories of the EAT-Lancet diet index					*P* value^2^
		≤ 13	14–16	17–19	20–22	≥ 23	
No. of participants	24,713	2364	5826	8788	5688	2047	–
Age (years)	58.0 ± 7.7	56.9 ± 7.2	57.7 ± 7.7	58.3 ± 7.8	58.5 ± 7.7	57.8 ± 7.6	< 0.0001
Sex (men, %)	9348 (37.8)	1418 (60.0)	2730 (46.9)	3210 (36.5)	1555 (27.3)	435 (21.3)	< 0.0001
Body mass index (kg/m^2^)	25.7 ± 4.0	25.4 ± 3.9	25.7 ± 4.0	25.9 ± 4.0	25.7 ± 3.9	25.2 ± 4.2	< 0.0001
University degree (%)	3608 (14.6)	234 (9.9)	763 (13.1)	1267 (14.4)	937 (16.5)	407 (19.9)	< 0.0001
Zero-consumers of alcohol (%)	1531 (6.2)	166 (7.0)	362 (6.2)	483 (5.5)	363 (6.4)	157 (7.7)	< 0.01
Leisure time physical activity (%)							
< 7.5	2365 (9.6)	389 (16.5)	668 (11.5)	776 (8.8)	397 (7.0)	135 (6.6)	< 0.0001
7.5–15	3615 (14.6)	420 (17.8)	932 (16.0)	1304 (14.8)	724 (12.7)	235 (11.5)	
15–25	5658 (22.9)	551 (23.3)	1378 (23.7)	2026 (23.1)	1296 (22.8)	407 (19.9)	
25–50	9010 (36.5)	695 (29.4)	1973 (33.9)	3273 (37.2)	2215 (38.9)	854 (41.7)	
> 50	4065 (16.5)	309 (13.1)	875 (15.0)	1409 (16.0)	1056 (18.6)	416 (20.3)	
Smoking status (%)							
Current	7034 (28.5)	1111 (47.0)	2038 (35.0)	2262 (25.7)	1210 (21.3)	413 (20.2)	< 0.0001
Former	8242 (33.4)	693 (29.3)	1830 (31.4)	3000 (34.1)	1975 (34.7)	744 (36.4)	
Never	9437 (38.2)	560 (23.7)	1958 (33.6)	3526 (40.1)	2503 (44.0)	890 (43.5)	
Diabetes (%)	1044 (4.2)	64 (2.7)	191 (3.3)	371 (4.2)	303 (5.3)	115 (5.6)	< 0.0001
Lipid-lowering medication (%)	619 (2.5)	23 (1.0)	120 (2.1)	229 (2.6)	195 (3.4)	52 (2.5)	< 0.0001
Antihypertensive drugs (%)	4124 (16.7)	294 (12.4)	917 (15.7)	1601 (18.2)	978 (17.2)	334 (16.3)	< 0.0001
Hypertension (%)	14,923 (60.4)	1382 (58.5)	3526 (60.5)	5392 (61.4)	3469 (61)	1154 (56.4)	< 0.001
Systolic blood pressure (mmHg)	141 ± 20	141 ± 20	141 ± 20	142 ± 20	141 ± 20	139 ± 20	< 0.0001
Diastolic blood pressure (mmHg)	85 ± 10	86 ± 10	86 ± 10	85 ± 10	85 ± 10	84 ± 10	< 0.0001
Total energy intake (kcal/day)	2265 ± 646	2654 ± 734	2413 ± 667	2234 ± 601	2094 ± 570	2007 ± 562	< 0.0001

^1^Continuous variables were expressed as means ± standard deviations and categorical variables as *n* (%)

^2^Chi-squared was used for categorical variables and one-way analysis of variance for continuous variables

During a median follow-up of 22.9 years (interquartile range: 16.2–24.7 years; maximum: 27.7 years; 493,460 person-years), a total of 4617 incident AF cases were documented. Table 2 presents the association between the EAT-Lancet diet index and the risk of AF. After adjusting for age, sex, dietary assessment version, season, and total energy intake (model 1), the highest adherence to the EAT-Lancet diet index (≥ 23 points) was associated with an 18% lower risk of AF (HR = 0.82; 95% CI: 0.71, 0.94; *P* for trend < 0.001) compared to the lowest adherence (≤ 13 points). Such associations were slightly attenuated but remained significant after further adjusting for leisure time physical activity, alcohol consumption, smoking status, and education (model 2); the multivariable HR (95% CI) of incident AF for the highest versus the lowest EAT-Lancet index categories was 0.85 (0.73, 0.98), *P* for trend < 0.01. Additional adjustments for BMI, diabetes, hypertension, and lipid-lowering medication did not appreciably change such associations (models 3 and 4).

**Table 2:** Association between the EAT-Lancet diet index and risk of atrial fibrillation in the Malmö Diet and Cancer Study (*n* = 24,713)^1^

	Categories of the EAT-Lancet diet index					*P* for trend^2^
	≤ 13	14–16	17–19	20–22	≥ 23	
Number of participants	2364	5826	8788	5688	2047	–
Number of cases	439	1122	1679	1029	348	–
Person-years	45,366	113,866	175,270	115,999	42,959	–
Incidence per 1000 person-years	9.68	9.85	9.58	8.87	8.10	–
Model 1	1.00 (reference)	0.96 (0.86, 1.08)	0.92 (0.83, 1.03)	0.85 (0.76, 0.95)	0.82 (0.71, 0.94)	< 0.001
Model 2	1.00 (reference)	0.98 (0.88, 1.09)	0.94 (0.85, 1.05)	0.87 (0.78, 0.98)	0.85 (0.73, 0.98)	< 0.01
Model 3	1.00 (reference)	0.97 (0.87, 1.08)	0.97 (0.87, 1.08)	0.87 (0.77, 0.98)	0.85 (0.74, 0.99)	< 0.01
Model 4	1.00 (reference)	0.96 (0.86, 1.07)	0.92 (0.82, 1.02)	0.85 (0.76, 0.96)	0.84 (0.73, 0.98)	< 0.01

Model 1: adjusted for age, sex, dietary assessment version (method), season, and total energy intake

Model 2: adjusted for variables in model 1 plus leisure time physical activity, alcohol consumption, smoking status, and educational level

Model 3: adjusted for variables in model 2 plus body mass index

Model 4: adjusted for variables in model 3 plus diabetes, hypertension, and lipid-lowering medication

^1^Values are given as hazard ratios and 95% confidence intervals within parentheses

^2^*P* for trend was calculated by assigning the categories of the EAT-Lancet diet index as the ordered categories

Table 3 presents the associations between individual components of the EAT-Lancet diet index and the risk of AF, adjusting for age, sex, dietary assessment version, season, total energy intake, leisure time physical activity, alcohol consumption, smoking status, education, BMI, diabetes, hypertension, and lipid-lowering medication. For the components of the EAT-Lancet diet index, higher intakes of vegetables and fruits as well as lower intakes of dairy and eggs were mainly driving the associations with AF.

**Table 3:** Associations between individual food components and risk of atrial fibrillation in the Malmö Diet and Cancer Study (*n* = 24,713)^1^

EAT-Lancet diet components	0	1	2	3	*P* for trend^2^
**Whole grains**	< 58 g/day	58–116 g/day	116–232 g/day	> 232 g/day	–
Multivariable model	1.00 (reference)	0.97 (0.91, 1.04)	1.02 (0.93, 1.10)	0.98 (0.82, 1.16)	0.97
**Potatoes**	> 200 g/day	100–200 g/day	50–100 g/day	< 50 g/day	–
Multivariable model	1.00 (reference)	0.92 (0.83, 1.00)	0.97 (0.88, 1.08)	0.98 (0.86, 1.12)	0.55
**Vegetables**	< 100 g/day	100–200 g/day	200–300 g/day	> 300 g/day	–
Multivariable model	1.00 (reference)	0.97 (0.90, 1.04)	0.94 (0.86, 1.03)	0.91 (0.80, 1.03)	0.08
**Fruits**	< 50 g/day	50–100 g/day	100–200 g/day	> 200 g/day	–
Multivariable model	1.00 (reference)	0.93 (0.82, 1.05)	0.91 (0.81, 1.02)	0.89 (0.80, 1.00)	0.07
**Dairy**	> 1000 g/day	500–1000 g/day	250–500 g/day	< 250 g/day	–
Multivariable model	1.00 (reference)	0.86 (0.79, 0.94)	0.91 (0.82, 1.01)	0.82 (0.70, 0.97)	0.09
**Beef and lamb**	> 28 g/day	14–28 g/day	7–14 g/day	< 7 g/day	–
Multivariable model	1.00 (reference)	0.97 (0.90, 1.05)	1.02 (0.91, 1.15)	1.06 (0.92, 1.22)	0.62
**Pork**	> 28 g/day	14–28 g/day	7–14 g/day	< 7 g/day	–
Multivariable model	1.00 (reference)	1.01 (0.92, 1.11)	0.95 (0.79, 1.13)	1.00 (0.83, 1.21)	0.86
**Poultry**	> 116 g/day	58–116 g/day	29–58 g/day	< 29 g/day	–
Multivariable model	1.00 (reference)	1.00 (0.62, 1.62)	0.98 (0.61, 1.56)	0.95 (0.60, 1.51)	0.28
**Eggs**	> 50 g/day	25–50 g/day	13–25 g/day	< 13 g/day	–
Multivariable model	1.00 (reference)	0.88 (0.79, 0.98)	0.82 (0.73, 0.91)	0.83 (0.74, 0.92)	< 0.01
**Fish**	< 7 g/day	7–14 g/day	14–28 g/day	> 28 g/day	–
Multivariable model	1.00 (reference)	1.04 (0.89, 1.22)	1.04 (0.92, 1.18)	1.04 (0.94, 1.16)	0.57
**Legumes**	< 18.75 g/day	18.75–37.5 g/day	37.5–75 g/day	> 75 g/day	–
Multivariable model	1.00 (reference)	0.96 (0.87, 1.07)	1.04 (0.81, 1.35)	0.85 (0.27, 2.62)	0.67
**Nuts**	< 12.5 g/day	12.5–25 g/day	25–50 g/day	> 50 g/day	–
Multivariable model	1.00 (reference)	0.93 (0.76, 1.15)	0.89 (0.62, 1.27)	0.80 (0.30, 2.13)	0.31
**Unsaturated oils**	< 10 g/day	10–20 g/day	20–40 g/day	> 40 g/day	–
Multivariable model	1.00 (reference)	1.08 (0.99, 1.18)	1.03 (0.95, 1.12)	0.99 (0.91, 1.08)	0.52
**Added sugar**	> 124 g/day	62–124 g/day	31–62 g/day	< 31 g/day	–
Multivariable model	1.00 (reference)	0.97 (0.83, 1.14)	0.94 (0.80, 1.11)	0.99 (0.82, 1.19)	0.95

Multivariable Cox models adjusted for age, sex, dietary assessment version (method), season, total energy intake, leisure time physical activity, alcohol consumption, smoking status, educational level, body mass index, diabetes, hypertension, and lipid-lowering medication. The results were based on multiple models (covariates and one individual component at a time)

^1^Values are given as hazard ratios and 95% confidence intervals within parentheses, with the respective 0 point group as the reference group

^2^*P* for trend was calculated by assigning the categories of the EAT-Lancet diet index as the ordered categories

In sensitivity analyses, the results were not substantially altered when participants with prevalent diabetes were excluded (Additional file 1: Table S2), when energy misreporters and individuals with eating habit changes were excluded (Additional file 1: Table S3), and when excluding AF cases within the first 2 or 5 years of follow-up (Additional file 1: Table S4). In subgroup analyses, there was no evidence of any effect modification by sex, age, BMI, leisure time physical activity, alcohol habits, education level, and smoking status (all *P* for interaction ≥ 0.32, Additional file 1: Table S5).

**Fig. 2:**
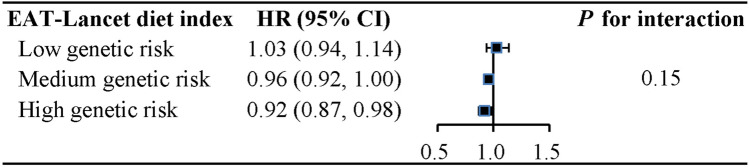
Association between the EAT-Lancet diet index and risk of atrial fibrillation according to genetic predisposition. The multivariable Cox proportional hazards regression model included age, sex, dietary assessment version (method), season, total energy intake, leisure time physical activity, alcohol consumption, smoking status, educational level, body mass index, diabetes, hypertension, and lipid-lowering medication. *P* for interaction was calculated by adding the cross-product term of the EAT-Lancet diet index and genetic risk score in the multivariable Cox model. HR, hazard ratio; CI, confidence interval

The age- and sex-adjusted HR (95% CI) of AF for individuals with high genetic risk was 2.76 (2.49, 3.05) compared to those with low genetic risk (Additional file 1: Table S6). Figure 2 shows the results stratified by categories of AF-GRS. The adjusted HRs (95% CIs) of AF per one SD increment of the EAT-Lancet diet index were 1.03 (0.94, 1.14), 0.96 (0.92, 1.00), and 0.92 (0.87, 0.98) among participants with low, medium, and high genetic risk, respectively (*P* for interaction = 0.15).

## Discussion

In this large prospective cohort of Swedish adults followed up for a median of 22.9 years and up to nearly 3 decades, we found that adherence to the EAT-Lancet diet index was associated with a lower risk of AF. Such association tended to be stronger in participants with higher genetic risk, albeit with no statistically significant interaction.

The current study is the first to investigate and generate evidence on the inverse association between the EAT-Lancet diet index and the risk of AF. Previous studies have shown protective associations between the EAT-Lancet reference diet (defined by using different scoring methods) and risks of ischemic heart disease, type 2 diabetes, stroke, and all-cause mortality [15, 30–32]. Our current study extends the potentially protective associations with the EAT-Lancet diet to AF. The findings were supported by previous studies where plant-based diets have been shown to reduce the risk of traditional AF-related risk factors [33]. In contrast, three prospective cohort studies did not observe a significant inverse association between a healthy dietary pattern, defined by the American Heart Association’s Life Simple 7 (including 5 items: fruits and vegetables, fish, whole grains, sodium, and sugar-sweetened beverages), and risk of AF [6, 10, 11]. Another prospective cohort study conducted in the US postmenopausal women indicated that adherence to the Portfolio Diet, as assessed by plant protein, nuts, viscous fiber, phytosterols, monounsaturated fatty acids, and saturated fat/cholesterol sources, was not significantly associated with an increased risk of AF (comparing the extreme quartiles: HR = 1.10, 95% CI: 0.87, 1.38) [9]. Furthermore, results from the Atherosclerosis Risk in Communities Study showed that low-carbohydrate diets were associated with an increased risk of incident AF [7]. Moreover, an age- and sex-matched case–control study (400 cases and 400 controls) indicated that patients with low adherence to the Mediterranean diet were more likely to have AF [8]. Similarly, another case–control study (68 cases and 85 controls) documented that adherence to the Mediterranean diet was inversely associated with the presence of AF (odds ratio: 0.65, 95% CI: 0.47, 0.91) [12].

In the current study, we for the first time assessed whether the association between diet and risk of AF is modified by genetic predisposition to AF. Although we did not observe a significant interaction, this association tended to be stronger in individuals with high genetic risk; in the low genetic risk group, the null association was likely to be due to the small sample size because the range of 95% CI was larger than the medium and high-risk groups. This phenomenon was supported by previous studies that indicated the beneficial effect of healthy dietary patterns on adverse health outcomes was more prominent in people with high genetic risk [34, 35]. Furthermore, this non-significant interaction may be due to the low percentage of the genetic risk explained by the SNPs included. In addition, different types of AF (e.g., permanent/paroxysmal AF) may be driven by distinct panels of SNPs. However, data on permanent/paroxysmal AF were not available in this study.

Among the EAT-Lancet diet index components, high intakes of vegetables and fruits were associated with a lower risk of AF, while low intakes of dairy and eggs were associated with a lower risk of AF, albeit no linear dose–response relationship of dairy and egg consumption with AF risk. The findings suggest that the potential beneficial association between the EAT-Lancet diet and AF may be largely driven by high intakes of vegetables and fruits and low intakes of dairy and eggs. The reason behind a low intake of dairy and eggs with a lower risk of AF may be due to the high intake of dairy, and eggs increase intake of cholesterol and saturated fatty acids. To our knowledge, no previous study examined these dietary factors in relation to the risk of AF. Therefore, future research needs to confirm such associations in other populations. Furthermore, considerably lower risks of AF were seen for the EAT-Lancet diet than for its components, suggesting that adherence to the EAT-Lancet diet index may benefit more than its components in preventing AF.

To the best of our knowledge, this was the first study that examined the association between the EAT-Lancet diet index and the risk of AF considering genetic susceptibility. The strengths of this study include a large-scale prospective population design with a long-term follow-up and a very low loss to follow-up (< 1%), validated measurements of diet and lifestyle data, and a reliable registry system for AF.

Nevertheless, this study has several limitations. First, dietary and other data were self-reported, and this may lead to measurement bias. However, such bias was most likely to be nondifferential misclassification, attenuating the association towards the null. Second, dietary data were collected only at baseline; thus, dietary habits might change over time during follow-up. However, some evidence indicates that diet in adulthood is relatively stable over time and measuring food habits once can be used to assess the effects of dietary patterns on future health outcomes [36]. In addition, the assessment of covariates was done only once at baseline. Information on covariates may change during the long follow-up period, therefore biasing the research findings. Third, the EAT-Lancet diet index is new and developed in the same data set. Thus, further validation of the diet index in a separate dataset would be crucial to increase confidence in the results. Fourth, it is not possible to differentiate AF and AFL in this study because AF cases were identified by the ICD-9 code 427.3 and the ICD-10 code I48.9, which included both AF and atrial AFL. Thus, we have decided to combine AF and AFL into one category, even though the pathogenesis for the two conditions partly differs. Fifth, although analyses were adjusted for a wide range of confounding factors, it is possible that unmeasured confounding remained. Sixth, the causality cannot be inferred from the observational study design. Finally, the present study only included a Swedish population; thus, further studies are necessary to assess the generalizability of the results to other populations.

## Conclusions

In conclusion, higher adherence to the EAT-Lancet diet was associated with a lower risk of AF among Swedish adults. Our results demonstrate that the recommendations for a healthy and sustainable diet proposed by the EAT-Lancet Commission may have beneficial effects on human health.

## Supplementary Information

**Additional file 1:**  **Table S1.** SNP list used to construct the genetic risk score of atrial fibrillation. **Table S2.** Association between the EAT-Lancet diet index and risk of atrial fibrillation in the Malmö Diet and Cancer Study, excluding participants with prevalent diabetes at baseline. **Table S3.** Association between the EAT-Lancet diet index and risk of atrial fibrillation in the Malmö Diet and Cancer Study, excluding energy mis-reporters and those with significant diet change. **Table S4.** Association between the EAT-Lancet diet index and risk of atrial fibrillation in the Malmö Diet and Cancer Study, excluding atrial fibrillation cases ascertained within the first two or five years of follow-up. **Table S5.** Association between the EAT-Lancet diet index and risk of atrial fibrillation in the Malmö Diet and Cancer Study, stratified by main baseline characteristics of participants. **Table S6.** Association between genetic risk score and risk of atrial fibrillation in the Malmö Diet and Cancer Study. **Fig. S1.** Distribution of the geneticrisk score of atrial fibrillation in the population.

## Data Availability

The data that support the findings of this study are available from “The Malmö Cohorts” at Lund University with the permission of the MDC Steering Committee (https://www.malmo-kohorter.lu.se/malmo-cohorts).

## Abbreviations

AF: Atrial fibrillation
AFL: Atrial flutter
BMI: Body mass index
CI: Confidence interval
FFQ: Food frequency questionnaire
GRS: Genetic risk score
HR: Hazard ratio
MDCS: Malmo Diet and Cancer Study
MET: Metabolic equivalent task
SD: Standard deviation
SNP: Single nucleotide polymorphism
STROBE: Strengthening the Reporting of Observational Studies in Epidemiology
