# Supplementary material for: The EAT-Lancet diet, genetic susceptibility and risk of atrial fibrillation in a population-based cohort
Source: BMC Med. 2023 Jul 28;21:280. doi: 10.1186/s12916-023-02985-6 (PMC10386230; doi:10.1186/s12916-023-02985-6)
Supplement: Supplementary file 1 — Additional file 1: Table S1. SNP list used to construct the genetic risk score of atrial fibrillation. Table S2. Association between the EAT-Lancet diet index and risk of atrial fibrillation in the Malmö Diet and Cancer Study, excluding participants with prevalent diabetes at baseline. Table S3. Association between the EAT-Lancet diet index and risk of atrial fibrillation in the Malmö Diet and Cancer Study, excluding energy mis-reporters and those with significant diet change. Table S4. Association between the EAT-Lancet diet index and risk of atrial fibrillation in the Malmö Diet and Cancer Study, excluding atrial fibrillation cases ascertained within the first two or five years of follow-up. Table S5. Association between the EAT-Lancet diet index and risk of atrial fibrillation in the Malmö Diet and Cancer Study, stratified by main baseline characteristics of participants. Table S6. Association between genetic risk score and risk of atrial fibrillation in the Malmö Diet and Cancer Study. Fig. S1. Distribution of the geneticrisk score of atrial fibrillation in the population. [file 12916_2023_2985_MOESM1_ESM.docx]

**Additional file for the manuscript:**

**Title:** The EAT-Lancet diet, genetic susceptibility and risk of atrial fibrillation in a population-based cohort

**Running Title:** EAT-Lancet diet, genetic susceptibility, and atrial fibrillation

**Author Names:** Shunming Zhang ^1,2*^, Anna Stubbendorff ^2^, Ulrika Ericson ^3^, Per Wändell ^4^, Kaijun Niu ^5^, Lu Qi ^6,7^, Yan Borné ^2#^, Emily Sonestedt ^2#*^

**Author Affiliations:** ^1^ School of Public Health, Xi’an Jiaotong University Health Science Center, Xi’an, Shaanxi, China.

^2^ Nutritional Epidemiology, Department of Clinical Sciences Malmö, Lund University, Malmö, Sweden.

^3^ Diabetes and Cardiovascular Disease-Genetic Epidemiology, Department of Clinical Sciences Malmö, Lund University, Malmö, Sweden.

^4^ Department of Neurobiology Care Sciences and Society, Division of Family Medicine and Primary Care, Karolinska Institutet, Huddinge, Sweden.

^5^ Nutritional Epidemiology Institute and School of Public Health, Tianjin Medical University, Tianjin, China.

^6^ Department of Epidemiology, School of Public Health and Tropical Medicine, Tulane University, New Orleans, LA, USA.

^7^ Department of Nutrition, Harvard T.H. Chan School of Public Health, Boston, MA, USA.

^#^These authors are joint senior authors.

***Correspondence to:**

Shunming Zhang, [shunming.zhang@med.lu.se](mailto:shunming.zhang@med.lu.se) and Emily Sonestedt, [emily.sonestedt@med.lu.se](mailto:emily.sonestedt@med.lu.se)

Nutritional Epidemiology, Department of Clinical Sciences Malmö, Lund University, Jan Waldenströms gata 35, 21428 Malmö, Sweden.

| **Table S1.** SNP list used to construct the genetic risk score of atrial fibrillation ^1^ | | | | | |
| --- | --- | --- | --- | --- | --- |
| **SNP** | **Chromosome** | **Risk/Ref Allele** | **RR** | **95% CI** | ***P* value** |
| rs187585530 | 1 | A/G | 1.55 | 1.36-1.77 | 1.18E-10 |
| rs880315 | 1 | C/T | 1.04 | 1.03-1.06 | 5.04E-09 |
| rs7529220 | 1 | C/T | 1.04 | 1.02-1.06 | 1.00E-04 |
| rs2885697 | 1 | G/T | 1.04 | 1.02-1.06 | 1.77E-07 |
| rs11590635 | 1 | A/G | 1.12 | 1.06-1.19 | 5.28E-05 |
| rs146518726 | 1 | A/G | 1.18 | 1.12-1.24 | 2.05E-10 |
| rs12044963 | 1 | T/G | 1.08 | 1.06-1.11 | 1.61E-12 |
| rs4484922 | 1 | G/C | 1.07 | 1.05-1.08 | 4.57E-16 |
| rs79187193 | 1 | G/A | 1.12 | 1.08-1.16 | 8.07E-10 |
| rs11264280 | 1 | T/C | 1.14 | 1.12-1.15 | 4.60E-59 |
| rs72700114 | 1 | C/G | 1.22 | 1.19-1.26 | 7.32E-48 |
| rs608930 | 1 | G/T | 1.1 | 1.09-1.12 | 1.94E-42 |
| rs10753933 | 1 | T/G | 1.08 | 1.06-1.09 | 5.83E-25 |
| rs4951261 | 1 | C/A | 1.05 | 1.03-1.06 | 1.17E-09 |
| rs6546620 | 2 | C/T | 1.07 | 1.05-1.09 | 2.96E-14 |
| rs6742276 | 2 | A/G | 1.05 | 1.03-1.06 | 2.42E-11 |
| rs2540949 | 2 | A/T | 1.08 | 1.06-1.09 | 8.17E-25 |
| rs10165883 | 2 | C/T | 1.07 | 1.05-1.08 | 5.83E-19 |
| rs72926475 | 2 | G/A | 1.07 | 1.05-1.1 | 3.49E-10 |
| rs28387148 | 2 | T/C | 1.06 | 1.03-1.09 | 7.35E-06 |
| rs67969609 | 2 | G/C | 1.06 | 1.03-1.09 | 5.10E-06 |
| rs12992412 | 2 | T/A | 1.04 | 1.03-1.06 | 2.30E-08 |
| rs56181519 | 2 | C/T | 1.08 | 1.06-1.1 | 1.52E-19 |
| rs35504893 | 2 | T/C | 1.09 | 1.08-1.11 | 6.89E-25 |
| rs295114 | 2 | C/T | 1.07 | 1.05-1.09 | 1.76E-20 |
| rs35544454 | 2 | A/T | 1.05 | 1.03-1.07 | 1.53E-06 |
| rs6810325 | 3 | C/T | 1.08 | 1.06-1.09 | 5.24E-23 |
| rs73032363 | 3 | A/G | 1.04 | 1.03-1.06 | 3.59E-08 |
| rs6790396 | 3 | G/C | 1.07 | 1.05-1.08 | 4.13E-18 |
| rs2306272 | 3 | C/T | 1.05 | 1.04-1.07 | 4.54E-11 |
| rs17005647 | 3 | T/C | 1.03 | 1.01-1.04 | 1.94E-04 |
| rs7632427 | 3 | T/C | 1.04 | 1.03-1.06 | 1.10E-08 |
| rs17490701 | 3 | G/A | 1.07 | 1.05-1.1 | 5.43E-11 |
| rs1278493 | 3 | G/A | 1.03 | 1.02-1.05 | 1.59E-05 |
| rs4855075 | 3 | T/C | 1.06 | 1.04-1.08 | 4.00E-09 |
| rs60902112 | 3 | T/C | 1.04 | 1.03-1.06 | 6.09E-07 |
| rs9872035 | 3 | C/T | 1.04 | 1.03-1.06 | 1.80E-08 |
| rs3822259 | 4 | T/G | 1.05 | 1.03-1.06 | 1.93E-09 |
| rs1458038 | 4 | T/C | 1.04 | 1.02-1.05 | 6.79E-06 |
| rs3960788 | 4 | C/T | 1.05 | 1.04-1.07 | 2.09E-12 |
| rs2129977 | 4 | A/G | 1.49 | 1.47-1.52 | 3.09e-525 |
| rs55754224 | 4 | T/C | 1.05 | 1.03-1.07 | 9.25E-09 |
| rs10213171 | 4 | G/C | 1.11 | 1.08-1.14 | 6.09E-14 |
| rs10520260 | 4 | A/G | 1.06 | 1.04-1.07 | 8.98E-12 |
| rs6596717 | 5 | C/A | 1.03 | 1.02-1.05 | 3.22E-06 |
| rs716845 | 5 | A/G | 1.06 | 1.04-1.08 | 1.16E-13 |
| rs2012809 | 5 | G/A | 1.05 | 1.03-1.07 | 1.98E-07 |
| rs34750263 | 5 | T/C | 1.09 | 1.08-1.11 | 2.89E-30 |
| rs174048 | 5 | C/T | 1.07 | 1.05-1.09 | 1.05E-11 |
| rs12188351 | 5 | A/G | 1.07 | 1.04-1.1 | 1.94E-05 |
| rs6882776 | 5 | G/A | 1.06 | 1.05-1.08 | 3.18E-14 |
| rs73366713 | 6 | G/A | 1.11 | 1.09-1.14 | 5.80E-21 |
| rs34969716 | 6 | A/G | 1.09 | 1.07-1.11 | 2.91E-25 |
| rs1307274 | 6 | T/G | 1.08 | 1.05-1.11 | 3.85E-08 |
| rs3176326 | 6 | G/A | 1.06 | 1.04-1.08 | 7.95E-11 |
| rs6907805 | 6 | G/T | 1.04 | 1.03-1.06 | 1.10E-08 |
| rs210632 | 6 | A/G | 1.05 | 1.03-1.07 | 2.75E-08 |
| rs17079881 | 6 | G/A | 1.09 | 1.07-1.11 | 4.23E-16 |
| rs13191450 | 6 | A/C | 1.07 | 1.06-1.09 | 8.92E-21 |
| rs12208899 | 6 | A/G | 1.05 | 1.03-1.07 | 1.95E-08 |
| rs117984853 | 6 | T/G | 1.12 | 1.09-1.15 | 8.38E-17 |
| rs11768850 | 7 | T/C | 1.04 | 1.03-1.05 | 4.96E-08 |
| rs55734480 | 7 | A/G | 1.05 | 1.03-1.07 | 7.34E-10 |
| rs6462078 | 7 | A/C | 1.06 | 1.04-1.08 | 1.35E-11 |
| rs74910854 | 7 | G/A | 1.1 | 1.07-1.13 | 3.36E-09 |
| rs11773884 | 7 | A/G | 1.05 | 1.03-1.07 | 4.72E-09 |
| rs62483627 | 7 | A/G | 1.05 | 1.03-1.07 | 5.17E-09 |
| rs11773845 | 7 | A/C | 1.12 | 1.11-1.14 | 4.61E-58 |
| rs55985730 | 7 | G/T | 1.1 | 1.06-1.14 | 1.81E-08 |
| rs7789146 | 7 | G/A | 1.06 | 1.04-1.08 | 6.51E-10 |
| rs35620480 | 8 | C/A | 1.05 | 1.03-1.07 | 1.01E-06 |
| rs7508 | 8 | A/G | 1.07 | 1.06-1.09 | 2.22E-19 |
| rs7846485 | 8 | C/A | 1.09 | 1.07-1.12 | 3.71E-15 |
| rs62521286 | 8 | G/A | 1.13 | 1.1-1.16 | 1.24E-16 |
| rs35006907 | 8 | A/C | 1.05 | 1.03-1.06 | 2.76E-09 |
| rs7460121 | 8 | A/G | 1.07 | 1.05-1.1 | 1.65E-08 |
| rs6993266 | 8 | A/G | 1.05 | 1.03-1.06 | 9.73E-10 |
| rs4977397 | 9 | A/G | 1.04 | 1.03-1.06 | 8.60E-09 |
| rs4385527 | 9 | A/G | 1.1 | 1.08-1.11 | 2.26E-36 |
| rs4743034 | 9 | A/G | 1.05 | 1.03-1.07 | 3.98E-09 |
| rs10760361 | 9 | G/T | 1.04 | 1.03-1.06 | 7.03E-09 |
| rs2274115 | 9 | G/A | 1.04 | 1.02-1.05 | 1.78E-05 |
| rs7919685 | 10 | G/T | 1.06 | 1.04-1.07 | 5.00E-16 |
| rs7096385 | 10 | T/C | 1.06 | 1.03-1.08 | 1.52E-05 |
| rs60212594 | 10 | G/C | 1.12 | 1.09-1.14 | 6.48E-27 |
| rs11001667 | 10 | G/A | 1.06 | 1.05-1.08 | 1.06E-11 |
| rs1044258 | 10 | T/C | 1.05 | 1.03-1.06 | 1.07E-09 |
| rs11598047 | 10 | G/A | 1.17 | 1.14-1.19 | 4.83E-58 |
| rs10749053 | 10 | T/C | 1.06 | 1.03-1.08 | 1.84E-07 |
| rs1822273 | 11 | G/A | 1.07 | 1.05-1.09 | 8.99E-17 |
| rs949078 | 11 | C/T | 1.05 | 1.04-1.07 | 4.77E-11 |
| rs76097649 | 11 | A/G | 1.13 | 1.1-1.17 | 2.19E-20 |
| rs10842383 | 12 | C/T | 1.11 | 1.09-1.14 | 1.02E-25 |
| rs113819537 | 12 | C/G | 1.05 | 1.03-1.07 | 2.23E-09 |
| rs12809354 | 12 | C/T | 1.08 | 1.06-1.11 | 5.48E-16 |
| rs7978685 | 12 | T/C | 1.06 | 1.04-1.07 | 5.99E-12 |
| rs35349325 | 12 | T/C | 1.05 | 1.04-1.07 | 9.04E-13 |
| rs11180703 | 12 | G/A | 1.05 | 1.03-1.06 | 3.58E-10 |
| rs883079 | 12 | T/C | 1.13 | 1.11-1.14 | 1.26E-51 |
| rs12810346 | 12 | T/C | 1.07 | 1.05-1.09 | 2.34E-09 |
| rs10773657 | 12 | C/A | 1.06 | 1.03-1.08 | 4.22E-07 |
| rs12298484 | 12 | C/T | 1.05 | 1.03-1.06 | 2.05E-09 |
| rs6560886 | 12 | C/T | 1.04 | 1.02-1.06 | 3.13E-05 |
| rs9580438 | 13 | C/T | 1.06 | 1.04-1.07 | 1.01E-13 |
| rs35569628 | 13 | T/C | 1.04 | 1.02-1.05 | 3.00E-05 |
| rs28631169 | 14 | T/C | 1.07 | 1.05-1.09 | 3.80E-14 |
| rs2145587 | 14 | A/G | 1.08 | 1.06-1.1 | 2.32E-21 |
| rs73241997 | 14 | T/C | 1.07 | 1.05-1.1 | 1.10E-13 |
| rs2738413 | 14 | A/G | 1.08 | 1.07-1.1 | 1.81E-29 |
| rs74884082 | 14 | C/T | 1.05 | 1.03-1.06 | 7.26E-08 |
| rs10873299 | 14 | A/G | 1.05 | 1.03-1.07 | 9.62E-11 |
| rs147301839 | 15 | C/A | 1.6 | 1.06-2.4 | 2.42E-02 |
| rs62011291 | 15 | G/A | 1.05 | 1.04-1.07 | 6.14E-09 |
| rs12591736 | 15 | G/A | 1.06 | 1.04-1.08 | 2.47E-09 |
| rs74022964 | 15 | T/C | 1.11 | 1.09-1.13 | 1.27E-27 |
| rs12908004 | 15 | G/A | 1.08 | 1.06-1.1 | 1.95E-14 |
| rs12908437 | 15 | T/C | 1.05 | 1.03-1.06 | 1.25E-10 |
| rs2286466 | 16 | G/A | 1.07 | 1.05-1.09 | 3.53E-14 |
| rs2359171 | 16 | A/T | 1.21 | 1.19-1.23 | 2.94E-100 |
| rs7225165 | 17 | G/A | 1.05 | 1.03-1.07 | 2.41E-05 |
| rs8073937 | 17 | G/A | 1.05 | 1.04-1.07 | 1.02E-11 |
| rs72811294 | 17 | G/C | 1.07 | 1.05-1.09 | 6.87E-09 |
| rs11658278 | 17 | T/C | 1.03 | 1.02-1.05 | 3.17E-06 |
| rs242557 | 17 | G/A | 1.04 | 1.03-1.06 | 4.35E-09 |
| rs76774446 | 17 | A/C | 1.07 | 1.04-1.09 | 1.13E-08 |
| rs7219869 | 17 | G/C | 1.05 | 1.03-1.06 | 1.49E-10 |
| rs12604076 | 17 | T/C | 1.02 | 1.01-1.04 | 1.28E-03 |
| rs9953366 | 18 | C/T | 1.05 | 1.04-1.07 | 9.03E-11 |
| rs8088085 | 18 | A/C | 1.03 | 1.02-1.05 | 5.15E-06 |
| rs2145274 | 20 | A/C | 1.11 | 1.08-1.14 | 6.97E-13 |
| rs7269123 | 20 | C/T | 1.05 | 1.03-1.06 | 5.59E-09 |
| rs2834618 | 21 | T/G | 1.12 | 1.09-1.14 | 2.93E-18 |
| rs465276 | 22 | G/A | 1.05 | 1.04-1.07 | 1.84E-11 |
| rs133902 | 22 | T/C | 1.04 | 1.03-1.06 | 1.06E-07 |
| ^1^ SNP, single nucleotide polymorphism; RR, relative risk; CI, confidence interval. | | | | | |

| **Table S2.** Association between the EAT-Lancet diet index and risk of atrial fibrillation in the Malmö Diet and Cancer Study, excluding participants with prevalent diabetes at baseline (n=23,669) ^1^ | | | | | | |
| --- | --- | --- | --- | --- | --- | --- |
|  | Categories of the EAT-Lancet diet index | | | | | *P* for trend ^2^ |
|  | ≤13 | 14-16 | 17-19 | 20-22 | ≥23 |  |
| Number of participants | 2,300 | 5,635 | 8,417 | 5,385 | 1,932 | - |
| Number of cases | 419 | 1,079 | 1,577 | 966 | 322 | - |
| Person-years | 44,324 | 110,768 | 169,120 | 110,688 | 40,890 | - |
| Incidence per 1000 person-years | 9.45 | 9.74 | 9.32 | 8.73 | 7.87 | - |
| Model 1 | 1.00 (reference) | 0.98 (0.87, 1.09) | 0.92 (0.82, 1.03) | 0.85 (0.76, 0.96) | 0.81 (0.70, 0.95) | <0.001 |
| Model 2 | 1.00 (reference) | 0.99 (0.88, 1.11) | 0.94 (0.84, 1.05) | 0.88 (0.78, 0.99) | 0.84 (0.72, 0.98) | <0.01 |
| Model 3 | 1.00 (reference) | 0.98 (0.87, 1.10) | 0.92 (0.82, 1.03) | 0.87 (0.77, 0.98) | 0.85 (0.73, 0.99) | <0.01 |
| Model 4 | 1.00 (reference) | 0.97 (0.87, 1.09) | 0.92 (0.82, 1.03) | 0.86 (0.77, 0.97) | 0.85 (0.73, 0.99) | <0.01 |
| ^1^ Values are given as hazard ratios and 95% confidence intervals within parentheses. | | | | | | |
| ^2^ *P* for trend was calculated by assigning the categories of the EAT-Lancet diet index as the ordered categories. | | | | | | |
| Model 1: adjusted for age, sex, dietary assessment version (method), season, and total energy intake. | | | | | | |
| Model 2: adjusted for variables in model 1 plus leisure-time physical activity, alcohol consumption, smoking status, and educational level. | | | | | | |
| Model 3: adjusted for variables in model 2 plus body mass index. | | | | | | |
| Model 4: adjusted for variables in model 3 plus hypertension and lipid-lowering medication. | | | | | | |

| **Table S3.** Association between the EAT-Lancet diet index and risk of atrial fibrillation in the Malmö Diet and Cancer Study, excluding energy mis-reporters and those with significant diet change (n=15,851) ^1^ | | | | | | |
| --- | --- | --- | --- | --- | --- | --- |
|  | Categories of the EAT-Lancet diet index | | | | | *P* for trend ^2^ |
|  | ≤13 | 14-16 | 17-19 | 20-22 | ≥23 |  |
| Number of participants | 1,707 | 4,173 | 5,784 | 3,266 | 921 | - |
| Number of cases | 319 | 811 | 1,092 | 590 | 154 | - |
| Person-years | 33,211 | 82,127 | 115,673 | 66,758 | 19,272 | - |
| Incidence per 1000 person-years | 9.61 | 9.87 | 9.44 | 8.84 | 7.99 | - |
| Model 1 | 1.00 (reference) | 0.97 (0.85, 1.11) | 0.90 (0.80, 1.03) | 0.84 (0.73, 0.96) | 0.78 (0.64, 0.95) | <0.001 |
| Model 2 | 1.00 (reference) | 0.98 (0.86, 1.12) | 0.92 (0.81, 1.04) | 0.86 (0.74, 0.99) | 0.80 (0.65, 0.97) | <0.01 |
| Model 3 | 1.00 (reference) | 0.97 (0.85, 1.11) | 0.90 (0.79, 1.03) | 0.86 (0.74, 0.99) | 0.80 (0.66, 0.98) | <0.01 |
| Model 4 | 1.00 (reference) | 0.97 (0.85, 1.11) | 0.90 (0.80, 1.03) | 0.86 (0.74, 0.99) | 0.80 (0.66, 0.98) | <0.01 |
| ^1^ Values are given as hazard ratios and 95% confidence intervals within parentheses. | | | | | | |
| ^2^ *P* for trend was calculated by assigning the categories of the EAT-Lancet diet index as the ordered categories. | | | | | | |
| Model 1: adjusted for age, sex, dietary assessment version (method), season, and total energy intake. | | | | | | |
| Model 2: adjusted for variables in model 1 plus leisure-time physical activity, alcohol consumption, smoking status, and educational level. | | | | | | |
| Model 3: adjusted for variables in model 2 plus body mass index. | | | | | | |
| Model 4: adjusted for variables in model 3 plus diabetes, hypertension, and lipid-lowering medication. | | | | | | |

| **Table S4.** Association between the EAT-Lancet diet index and risk of atrial fibrillation in the Malmö Diet and Cancer Study, excluding atrial fibrillation cases ascertained within the first two or five years of follow-up ^1^ | | | | | | |  |
| --- | --- | --- | --- | --- | --- | --- | --- |
|  | Categories of the EAT-Lancet diet index | | | | | *P* for trend ^2^ |  |
|  | ≤13 | 14-16 | 17-19 | 20-22 | ≥23 |  |  |
| Excluding cases within first 2 y | 1.00 (reference) | 0.96 (0.86, 1.08) | 0.92 (0.82, 1.02) | 0.86 (0.76, 0.97) | 0.85 (0.74, 0.99) | <0.01 |  |
| Excluding cases within first 5 y | 1.00 (reference) | 0.95 (0.85, 1.06) | 0.91 (0.81, 1.02) | 0.85 (0.76, 0.96) | 0.84 (0.72, 0.98) | <0.01 |  |
| ^1^ Values are given as hazard ratios and 95% confidence intervals within parentheses. | | | | | | |  |
| ^2^ *P* for trend was calculated by assigning the categories of the EAT-Lancet diet index as the ordered categories. | | | | | | |  |
| Multivariable Cox models adjusted for age, sex, dietary assessment version (method), season, total energy intake, leisure-time physical activity, alcohol consumption, smoking status, educational level, body mass index, diabetes, hypertension, and lipid-lowering medication. | | | | | | |  |
|  |  |  |  |  |  |  |  |

| **Table S5.** Association between the EAT-Lancet diet index and risk of atrial fibrillation in the Malmö Diet and Cancer Study, stratified by main baseline characteristics of participants (n=24,713) ^1^ | | | | | | | |
| --- | --- | --- | --- | --- | --- | --- | --- |
|  | Categories of the EAT-Lancet diet index | | | | | *P* for trend ^2^ | *P* for interaction ^3^ |
|  | ≤13 | 14-16 | 17-19 | 20-22 | ≥23 |  |  |
| Sex |  |  |  |  |  |  |  |
| Men (n=9,348) | 1.00 (reference) | 1.03 (0.90, 1.19) | 1.01 (0.88, 1.16) | 0.85 (0.72, 0.99) | 0.79 (0.62, 1.00) | <0.01 | 0.49 |
| Women (n=15,365) | 1.00 (reference) | 0.83 (0.68, 1.00) | 0.78 (0.65, 0.94) | 0.78 (0.64, 0.93) | 0.78 (0.63, 0.96) | 0.04 |  |
| Age (years) |  |  |  |  |  |  |  |
| <65 (n=19,242) | 1.00 (reference) | 0.98 (0.86, 1.11) | 0.90 (0.79, 1.02) | 0.86 (0.74, 0.99) | 0.87 (0.73, 1.04) | <0.01 | 0.32 |
| ≥65 (n=5,472) | 1.00 (reference) | 0.95 (0.76, 1.17) | 0.96 (0.78, 1.18) | 0.85 (0.68, 1.06) | 0.82 (0.62, 1.07) | 0.04 |  |
| Body mass index (kg/m^2^) |  |  |  |  |  |  |  |
| <25 (n=11,740) | 1.00 (reference) | 0.89 (0.74, 1.06) | 0.82 (0.69, 0.98) | 0.85 (0.70, 1.02) | 0.79 (0.63, 1.00) | 0.06 | 0.91 |
| ≥25 (n=12,973) | 1.00 (reference) | 1.00 (0.87, 1.16) | 0.97 (0.85, 1.12) | 0.86 (0.74, 1.00) | 0.86 (0.71, 1.04) | <0.01 |  |
| Leisure-time physical activity  (MET-hour/week) |  |  |  |  |  |  |  |
| <7.5 (n=2,365) | 1.00 (reference) | 0.93 (0.70, 1.25) | 0.88 (0.65, 1.18) | 0.63 (0.43, 0.90) | 0.85 (0.52, 1.37) | 0.04 | 0.84 |
| 7.5-15 (n=3,615) | 1.00 (reference) | 0.88 (0.66, 1.17) | 0.92 (0.70, 1.21) | 1.03 (0.76, 1.40) | 0.81 (0.53, 1.22) | 0.99 |  |
| 15-25 (n=5,658) | 1.00 (reference) | 0.87 (0.69, 1.10) | 0.82 (0.65, 1.02) | 0.82 (0.64, 1.04) | 0.75 (0.54, 1.04) | 0.07 |  |
| 25-50 (n=9,010) | 1.00 (reference) | 1.05 (0.87, 1.28) | 0.94 (0.77, 1.14) | 0.90 (0.73, 1.10) | 0.86 (0.68, 1.11) | 0.02 |  |
| >50 (n=4,065) | 1.00 (reference) | 1.02 (0.75, 1.37) | 1.09 (0.81, 1.45) | 0.84 (0.62, 1.14) | 1.01 (0.71, 1.43) | 0.27 |  |
| Alcohol habits |  |  |  |  |  |  |  |
| Zero-consumers (n=1,531) | 1.00 (reference) | 0.81 (0.52, 1.26) | 0.98 (0.64, 1.51) | 0.74 (0.47, 1.17) | 0.83 (0.48, 1.43) | 0.40 | 0.44 |
| Consumers (n=23,182) | 1.00 (reference) | 0.97 (0.86, 1.09) | 0.91 (0.82, 1.02) | 0.86 (0.76, 0.97) | 0.85 (0.73, 0.99) | <0.01 |  |
| University degree |  |  |  |  |  |  |  |
| Yes (n=3,608) | 1.00 (reference) | 0.82 (0.59, 1.16) | 0.89 (0.64, 1.23) | 0.75 (0.53, 1.06) | 0.79 (0.53, 1.19) | 0.21 | 0.52 |
| No (n=21,105) | 1.00 (reference) | 0.97 (0.86, 1.09) | 0.91 (0.81, 1.02) | 0.86 (0.76, 0.98) | 0.85 (0.73, 0.99) | <0.01 |  |
| Smoking status |  |  |  |  |  |  |  |
| Current (n=7,034) | 1.00 (reference) | 0.94 (0.78, 1.12) | 0.87 (0.72, 1.04) | 0.71 (0.57, 0.89) | 0.83 (0.62, 1.13) | <0.01 | 0.54 |
| Former (n=8,242) | 1.00 (reference) | 1.01 (0.83, 1.23) | 1.00 (0.83, 1.21) | 0.99 (0.81, 1.21) | 0.84 (0.65, 1.09) | 0.24 |  |
| Never (n=9,437) | 1.00 (reference) | 0.94 (0.76, 1.16) | 0.89 (0.73, 1.09) | 0.84 (0.68, 1.03) | 0.87 (0.68, 1.12) | 0.08 |  |
| ^1^ Values are given as hazard ratios and 95% confidence intervals within parentheses. | | | | | | | |
| ^2^ *P* for trend was calculated by assigning the categories of the EAT-Lancet diet index as the ordered categories. | | | | | | | |
| ^3^ *P* for interaction was calculated using the likelihood ratio test by comparing models with and without the interaction terms. | | | | | | | |
| Multivariable Cox models adjusted for age, sex, dietary assessment version (method), season, total energy intake, leisure-time physical activity, alcohol consumption, smoking status, educational level, body mass index, diabetes, hypertension, and lipid-lowering medication. | | | | | | | |

| **Table S6.** Association between genetic risk score and risk of atrial fibrillation in the Malmö Diet and Cancer Study (n=23,654) ^1^ | | | | |
| --- | --- | --- | --- | --- |
|  | Low genetic risk | Medium genetic risk | High genetic risk | *P* for trend |
| Model 1 | 1.00 (reference) | 1.69 (1.54, 1.85) | 2.62 (2.37, 2.90) | <0.0001 |
| Model 2 | 1.00 (reference) | 1.73 (1.57, 1.90) | 2.76 (2.49, 3.05) | <0.0001 |
| ^1^ Values are given as hazard ratios and 95% confidence intervals within parentheses. | | | | |
| Model 1 was unadjusted. | | | | |
| Model 2 was adjusted for age and sex. | | | | |


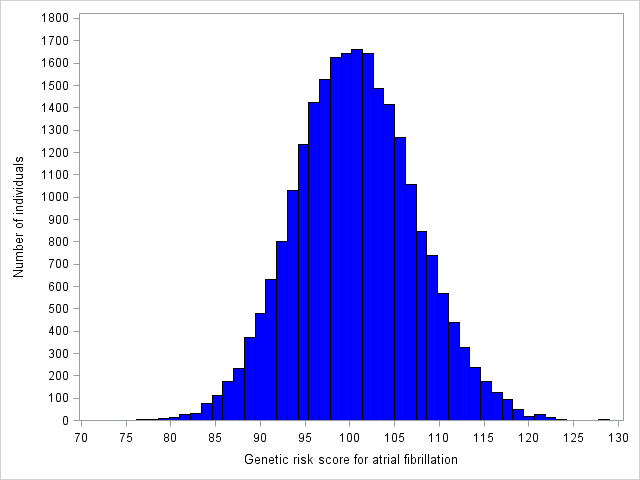


**Fig. S1.** Distribution of the genetic risk score of atrial fibrillation (range: 76.6 to 128.7) in the population (n=23,654).
